# Implementation of a National Measles Elimination Program in Iran: Phylogenetic Analysis of Measles Virus Strains Isolated during 2010–2012 Outbreaks

**DOI:** 10.1371/journal.pone.0094846

**Published:** 2014-04-15

**Authors:** Vahid Salimi, Simin Abbasi, Seyed Mohsen Zahraei, Ghazal Fatemi-Nasab, Fatemeh Adjaminezhad-Fard, Azadeh Shadab, Nastaran Ghavami, Raziyeh Zareh-Khoshchehre, Rambod Soltanshahi, Louis Bont, Talat Mokhtari-Azad

**Affiliations:** 1 Department of Virology, School of Public Health, Tehran University of Medical Sciences, Tehran, Iran; 2 National Reference Laboratory for Measles and Rubella, School of Public Health, Tehran University of Medical Sciences, Tehran, Iran; 3 Vaccine Preventable Diseases Department, Center for Communicable Diseases Control, Ministry of Health and Medical Education, Tehran, Iran; 4 Department of Pediatrics and Department of Immunology, University Medical Center Utrecht, Utrecht, the Netherlands; Fondazione Bruno Kessler, Italy

## Abstract

Measles virus (MV) causes small and large outbreaks in Iran. Molecular assays allow identifying and the sources of measles imported from neighboring countries. We carried out a phylogenetic analysis of measles virus circulating in Iran over the period 2010–2012. Specimens from suspected cases of measles were collected from different regions of Iran. Virus isolation was performed on urine and throat swabs. Partial nucleoprotein gene segments of MV were amplified by RT-PCR. PCR products of 173 samples were sequenced and analyzed. The median age of confirmed cases was 2 years. Among all confirmed cases, 32% had unknown vaccination status, 20% had been vaccinated, and 48% had not been vaccinated. Genotypes B3 and D8 (for the first time), H1 and D4 were detected mainly in unvaccinated toddlers and young children. Genotype B3 became predominant in 2012 and was closely related to African strains. H1 strains were also found in small and large outbreaks during 2012 but were not identical to Iranian H1-2009 strains. A majority of the Iranian D4 strains during 2010–2012 outbreaks were linked to the D4 strain identified in the Pakistan in 2007. We identified a single case in 2010 belonging to D8 genotype with 99.7% identity to Indian isolates. Although the vaccination program is currently good enough to prevent nationwide epidemics and successfully decreased measles incidence in Iran, the fraction of protected individuals in the population was not high enough to prevent continuous introduction of cases from abroad. Due to increasing number of susceptible individuals in some areas, sustained transmission of the newly introduced viral genotype remains possible.

## Introduction

Measles is an acute and highly transmissible respiratory infection caused by measles virus (MV). [1] Despite effective vaccination that resulted in a 71% drop in measles deaths between 2000 and 2011 worldwide, global mortality attributed to measles has been estimated to be 157700 deaths in 2011 [2]. Human MV belongs to the genus Morbillivirus within the family *Paramyxoviridae* and is widespread throughout the world [1]. The C-terminal 450 nucleotides of N-gene sequence is one of the most variable parts of the measles genome and commonly used for genetic characterization of wild-type viruses. Based on the analysis of this genomic region, there are 24 recognized genotypes within the 8 clades (A–H) [3]. Genotyping of wild type MV allows identifying imported measles as well as indigenous strains [4]. Before measles surveillance program in 1980, measles was endemic in all provinces of Iran. After introduction of single-shot monovalent measles vaccination in 1967, a two-dose strategy (monovalent measles vaccine at the ages of 9 months and 15 months) was introduced in 1987. A mass vaccination campaign for all persons aged 5–25 years in 2003 (two doses of measles and rubella (MR)) and follow up vaccination from 2004 onwards (two doses of measles, mumps, and rubella (MMR) plus vaccination at the ages of 12 months and 18 months, measles incidence in Iran has been declining steadily [5]. From 2002, National Measles Laboratory of Iran in collaboration with Center for Disease Control (CDC-Iran) conducts routine molecular surveillance of MV associated with outbreaks and sporadic cases [5], [6]. Small and large outbreaks have occurred periodically in Iran. Two different measles genotypes have been reported from 2002-May 2010 in Iran [7]. Genotype D4 was considered as the indigenous genotype before mass vaccination [8], [9] and genotype H1 as imported genotype from distant parts of Asia [7]. According to the new measles elimination strategy of the WHO, Iran aimed to eliminate MV by 2015 [2]. At present, a limited number of provinces in Iran are prone to measles outbreaks because of inappropriate adoption of the measles immunization program [6]. Since the disease is highly contagious we aimed to obtain insight into the molecular epidemiology of recent measles outbreaks in all provinces of Iran and compare virus strains to strains found in epidemics in other parts of the world. This information will help us to understand global transmission pattern of endemic measles.

## Methods

### Clinical Specimens

The study was carried out and approved by the ethical committee of ministry of health of Iran for the protection of human subjects in Iran and written informed consent was obtained from all persons or their parents for participation and sample collection. Nose-throat swabs, urine and serum specimens of measles suspected cases from 1^st^ May 2010 to 30^th^ December 2012 were collected from primary health care centers in different regions of Iran by Ministry of Health and Medical Education in Iran. All the specimens were collected 1–15 (average of 4.8) days after onset of rash according to WHO procedures [10] and transported to the National Measles Laboratory of Iran in the School of Public Health, Tehran University of Medical Science for processing.

### Antibody, RNA Detection and Viral Isolation

All sera were screened for measles IgM specific antibody using the commercial ELISA kits (Dade Behring, Marburg, Germany). Nose-throat swabs and urine of IgM confirmed cases were also processed and inoculated onto Vero/hSLAM cells [11]. Cells were harvested when CPE was observed and virus isolation was confirmed by RT-PCR. Confirmed cases were further analyzed by RT-PCR and bidirectional sequencing. If virus culture was not successful for direct detection of MV, confirmed cases were further analyzed by RT-PCR and sequencing. Total RNA was extracted from measles isolates (infected cell lysates) or directly from clinical specimens according to the manufacturer’s instructions with High Pure Viral Nucleic Acid Kit (Roche Diagnostics, Germany). Partial nucleoprotein gene segments (the C-terminus of N gene) of MV were amplified by RT-PCR using previously described primers MeV214 and MeV216 [12].

### Sequencing and Data Analysis

Sequencing was done on a capillary sequencer (ABI automatic DNA Analyzer, Life technologies) and the ABI BigDye Terminator V3.1 cycle sequencing kit (Life Technologies) according to the manufacturer’s instructions in both directions. Wild-type measles isolates and genotype sequences from Iran were named as recommended by WHO [13]. Genotyping of the sequences was performed according to WHO protocol [10]. All sequence comparisons were based on analysis of the 450 bp sequence. Phylogenetic analysis using MEGA version.5 [14] was based on the Neighbor Joining and Kimura 2-parameter methods and bootstrap analyses were performed by 1,000 resampling of the data sets. Sequences measles strains of other countries were downloaded from GenBank and Measles Nucleotide Surveillance database (MeaNS) (http://www.who-measles.org/) and included in the analysis. All sequences obtained during this study were submitted to the GenBank and MeaNS.

## Results

**Figure 1:**
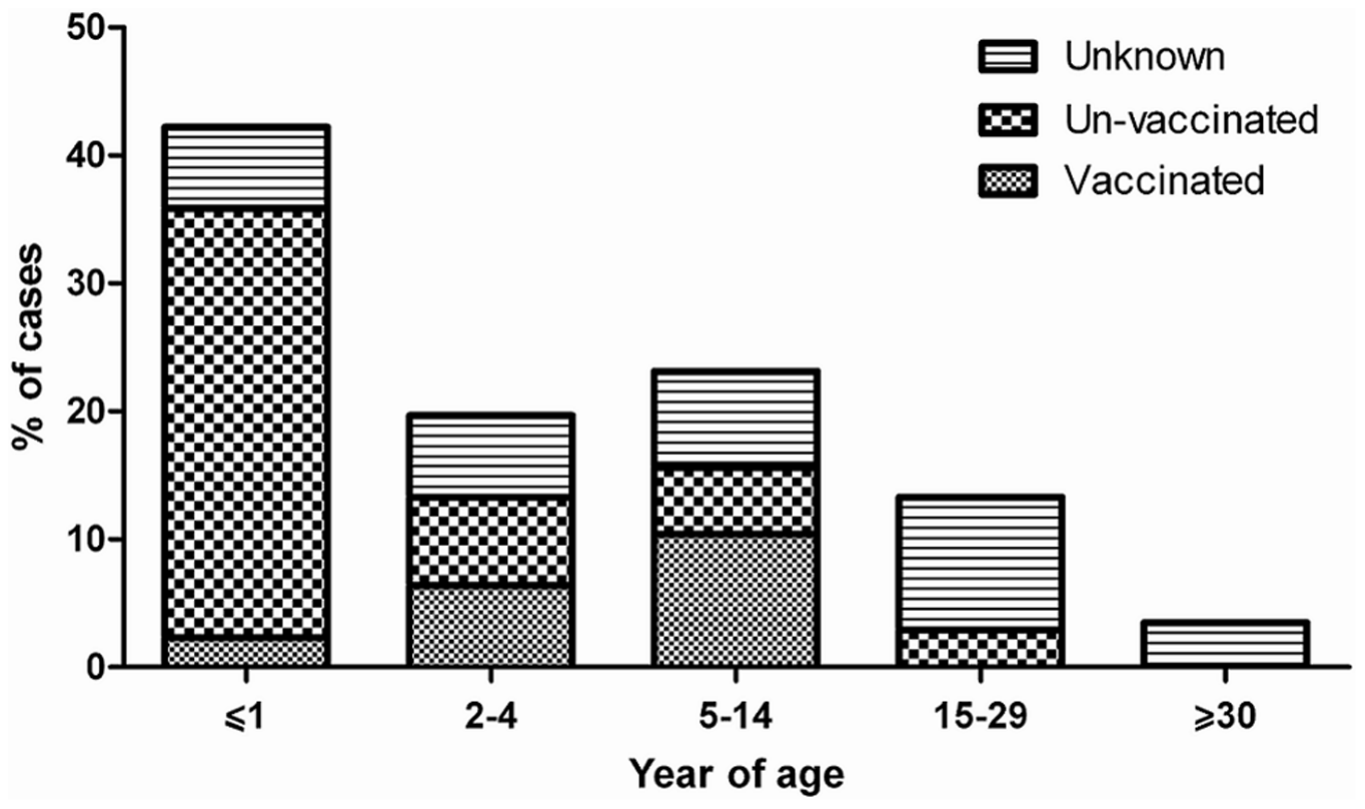
Measles, number of cases by age group and vaccination status, Iran, 2010–2012 (n = 173).

**Figure 2:**
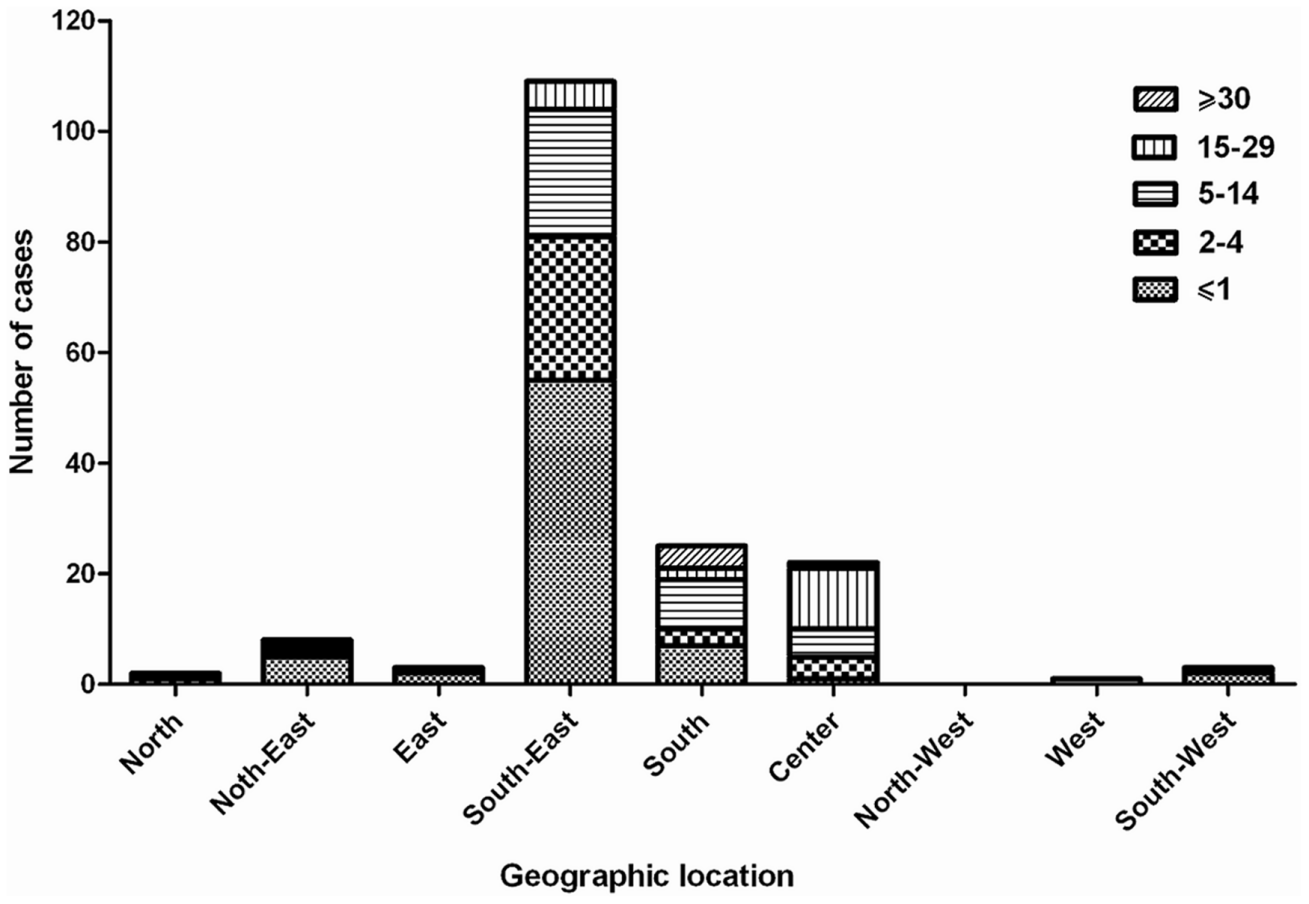
Age distribution of measles cases related to geographical location, Iran, 2010–2012 (n = 173).

In spite of progress in measles surveillance, outbreaks were reported from some parts of our country over the last 3 years. A total of 438 cases of measles (from 1^st^ May 2010–30^th^ December 2012) were confirmed by a serological test (IgM-ELISA). Case patients with measles symptoms who were IgM positive were considered confirmed cases. Sequence analysis was undertaken on 173 confirmed measles cases which were both serologically positive and positive by RT-PCR (Table S1). 44 sequences (25.4%) were obtained from viral isolates using Vero/hSLAM cell culture (21 cases isolated from throat swab and 23 from urine) and the 129 (74.6%) were obtained directly from clinical samples. We were able to isolate live MV from cases during all outbreaks, which is useful for a more extensive characterization of the strains involved in the outbreaks. Demographic information for the 173 sequences and the representative sequences are shown in the Table S1 and Table 1, respectively. The age of cases ranged from 1 month to 60 years (6.47±9.16 years). The median age was 2 years. 37.6% (65 cases) were female and 62.4% (108 cases) were male. The proportion of gender was significantly different as the male-to-female ratio was 1.66∶1. The age distribution of laboratory confirmed cases shows 61.8% of them were less than 5 years (Figure 1). Age distribution of measles cases related to geographical location shows that the susceptible population specially in patients less than 5 years is increasing in South and South-East of Iran (Figure 2). The vaccination history showed that among all 173 confirmed cases, 34 (20%) had been vaccinated, 83 (48%) had not been vaccinated, and 56 (32%) had unknown vaccination status (Table S1). 77% of unvaccinated cases were under 2 years old, who should have been vaccinated with two MMR doses. Six confirmed cases had traveled from Pakistan and Afghanistan (Table S1). Complete geographic distribution of measles cases are indicated in the Table S1. 69.4% of measles cases occurred in provinces in South-Eastern Iran that share borders with Afghanistan and Pakistan.

**Table 1:** Characteristics of the representative positive samples collected from 1^st^ May 2010 to 30^th^ December 2012 in Iran*.

NO	Strain Designation	Age(year)	Gender	Source ofspecimen	Genotype	Province oforigin	VaccineHistory	Accessionnumber
1	MVi/Bandarabas.IRN/5.10/1	9M†	F	Urine	D4	South	None	HM440226
4	MVi/Bandarlengeh.IRN/07.10/1	15	F	Urine	D4	South§	Unknown	HM440228
6	MVs/Sistan.IRN/10.10	4M	F	TS‡	D4	South East	None	HM998697
7	MVs/Tehran.IRN/14.10	20	M	TS	D4	Center¶	None	HM998698
8	MVs/Sistan.IRN/16.10/1	2	F	TS	D4	South East	Yes	HM998699
10	MVs/Sistan.IRN/17.10	6M	M	TS	D4	South East	None	HM998701
11	MVs/Sistan.IRN/18.10	4	M	TS	D4	South East	None	HM998702
12	MVi/Sistan.IRN/19.10/1	4	M	TS	D4	South East	Yes	HM998703
14	MVi/Sistan.IRN/20.10/1	2	M	TS	D4	South East	Yes	HM998705
16	MVi/Tehran.IRN/21.10/1	23	M	Urine	D4	Center	None	HM998707
19	MVs/Isfahan.IRN/22.10	5	M	TS	D4	Center	Yes	HQ395674
20	MVs/Sistan.IRN/23.10/1	1	F	TS	D4	South East	None	HQ395675
23	MVs/Isfahan.IRN/24.10	3	M	TS	D4	Center	Yes	HQ395677
24	MVs/Isfahan.IRN/25.10/1	8M	M	TS	D4	Center	None	HQ395678
26	MVs/Ghom.IRN/27.10/1	16	M	TS	D4	Center	Unknown	HQ395680
28	MVi/Hormozgan.IRN/28.10/1	31	F	Urine	D4	South	Unknown	HQ596506
30	MVs/Sistan.IRN/29.10	11M	M	TS	D4	South East	None	HQ395681
31	MVs/Mashhad.IRN/30.10/1	8M	F	TS	D4	North East	None	HQ395682
35	MVs/BandarJask.IRN/31.10/1	6	F	TS	D4	South	Yes	HQ395684
37	MVs/Fars.IRN/34.10	11M	F	TS	D4	South West	Unknown	HQ596510
38	MVs/Mashhad.IRN/35.10/1	8	F	Urine	D4	North East	Yes	HQ596511
41	MVs/Mashhad.IRN/39.10/1	8M	M	TS	D4	North East	Unknown	HQ596514
43	MVs/Mashhad.IRN/40.10/1	10M	M	TS	D4	North East	Unknown	HQ668019
45	MVs/Sistan.IRN/42.10	1M	M	TS	D4	South East¶	Unknown	HQ668021
46	MVs/Sistan.IRN/43.10	2	M	TS	D4	South East¶	Unknown	HQ668022
47	MVs/Sistan.IRN/45.10	2	M	TS	D4	South East	Unknown	HQ711619
48	MVs/Sistan.IRN/1.11	7	F	TS	D4	South East	Unknown	JF327854
49	MVs/Fars.IRN/5.11	7	F	TS	D4	South West	None	JF716447
50	MVi/Tehran.IRN/14.11/1	21	M	Urine	D4	Center	Unknown	JN048663
52	MVs/Hormozgan.IRN/18.11	1	F	Urine	D4	South	None	JN048665
53	MVs/Sistan.IRN/52.11	7	F	Urine	D4	South East	Unknown	JQ687376
54	MVi/Yazd.IRN/2.12	23	M	Urine	D4	Center	Unknown	JQ687377
55	MVs/Tehran.IRN/9.12	28	M	TS	B3	Center	Unknown	JX183266
56	MVi/Sistan.IRN/12.12/1	5	M	TS	B3	South East	Unknown	JX051516
58	MVs/Sistan.IRN/14.12/1	17	F	TS	B3	South East	None	JX183259
60	MVi/Sistan.IRN/15.12/1	11	M	Urine	B3	South East	None	JX183261
65	MVi/Sistan.IRN/16.12/1	5	M	TS	B3	South East	Yes	JX183267
72	MVi/Sistan.IRN/17.12/1	11M	M	TS	B3	South East	None	JX183274
75	MVs/Tehran.IRN/18.12/1	3	M	Urine	B3	Center	None	JX183276
81	MVi/Sistan.IRN/19.12/1	10	M	Urine	B3	South East	Unknown	JX219961
89	MVs/Sistan.IRN/20.12/1	5	M	TS	D4	South East	None	JX219968
100	MVs/Tehran.IRN/21.12/1	33	F	TS	B3	Center	Unknown	JX418027
111	MVi/Sistan.IRN/22.12/1	11M	M	TS	B3	South East	None	JX486009
115	MVs/Yazd.IRN/23.12/1	22	M	TS	H1	Center	Unknown	JX418032
119	MVs/Ghom.IRN/24.12/1	22	M	Urine	B3	Center	Unknown	JX486013
122	MVs/Sistan.IRN/25.12/1	14	M	Urine	B3	South East	Yes	JX486015
125	MVs/Sistan.IRN/26.12/1	10M	M	TS	B3	South East	None	JX631249
127	MVs/Sistan.IRN/27.12/1	3	M	TS	B3	South East	None	JX631251
137	MVs/Sistan.IRN/28.12/1	6M	F	TS	B3	South East	None	JX857307
142	MVs/Kerman.IRN/29.12/1	10	M	TS	B3	Center	Yes	JX857312
146	MVs/Sistan.IRN/30.12/1	2	M	TS	B3	South East	None	JX857314
148	MVs/Konarak.IRN/35.12/1	6M	M	Urine	B3	South East	None	KC139052
155	MVs/Chabahar.IRN/38.12/1	2.4	M	TS	B3	South East	Yes	KC139059
162	MVs/Chabahar.IRN/42.12	1.2	M	TS	B3	South East	None	KC879273
167	MVs/Iranshahr.IRN/43.12/1	1.2	F	TS	B3	South East	None	KF214765
168	MVi/Zabol.IRN/45.12/1	7	M	Urine	D4	East	Yes	KC984305
169	MVs/Chabahar.IRN/50.12/1	10M	M	TS	B3	South East	None	KC737545
172	MVs/Konarak.IRN/51.12	9M	M	TS	B3	South East	None	KC794528
173	MVs/Zahedan.IRN/52.12	1M	M	TS	B3	South East	None	KC794532

*The nomenclature indicates the representative sample which is the first isolated strain during that week.

† Month.

‡ Throat swab.

§ Pakistani who is imported cases.

¶ Afghanis who are imported cases.

### Phylogenetic Analysis of MV D4 Genotypes

**Figure 3:**
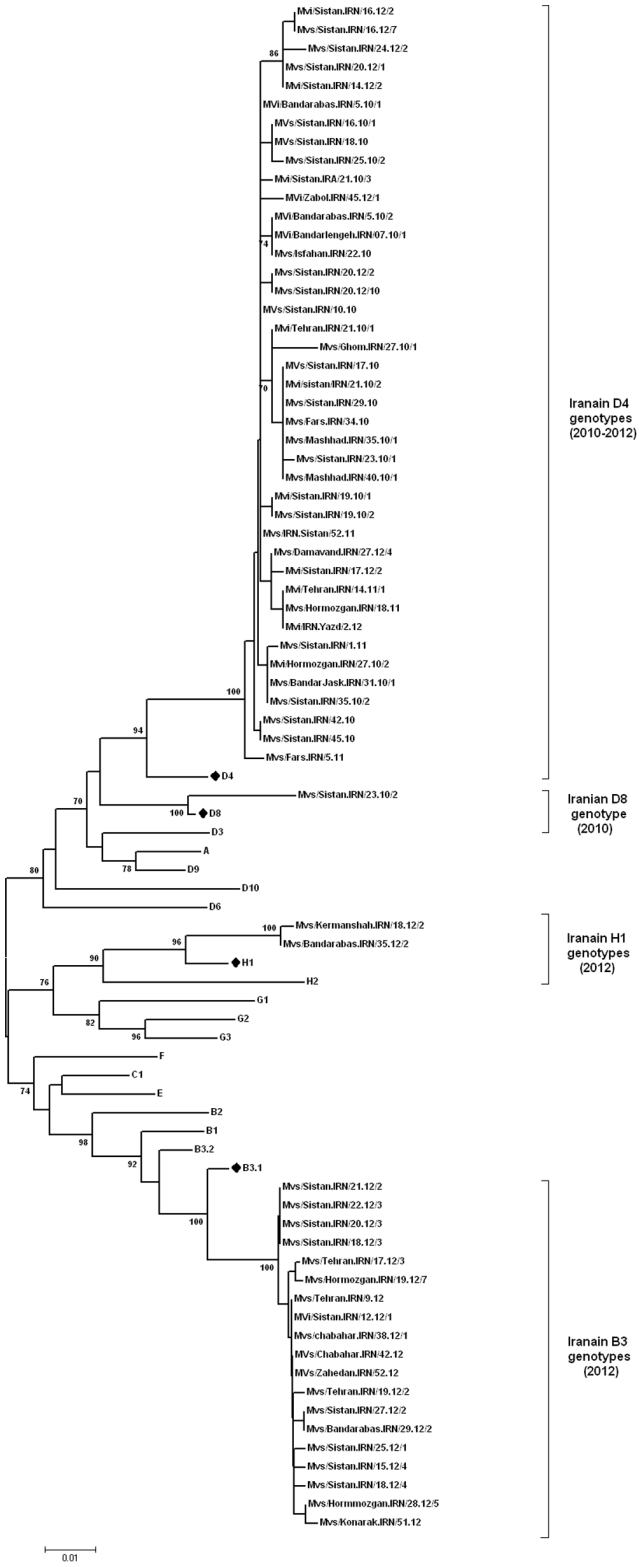
Phylogenetic tree of the C-terminus of N gene sequences of 63selected wild-type measles isolates from Iran compared to the reference sequences for each genotype. The Iranian sequences are belonged to B3, D8, D4 and H1 genotypes during 2010–2012. Bootstrap values (1,000 replicates) >70% are indicated. Closely related reference sequences to the Iranian strains are shown by dark rhombic. Scale bar indicates base substitutions per site. Accession numbers of WHO reference sequences are: Genotype A (U01987), B1 (U01998), B2 (U01994), B3.1 (AJ232203), B3.2 (L46753), C1 (AJ232203), D3 (U01977),D4 (U01976), D6 (L46750), D8 (AF280803), D9 (AF481485), D10 (AY923185), E (X84879), F(X84865), G1(U01974), G2(AF171232), G3(AY184217), H1(AF045212), H2(AF045217).

**Figure 4:**
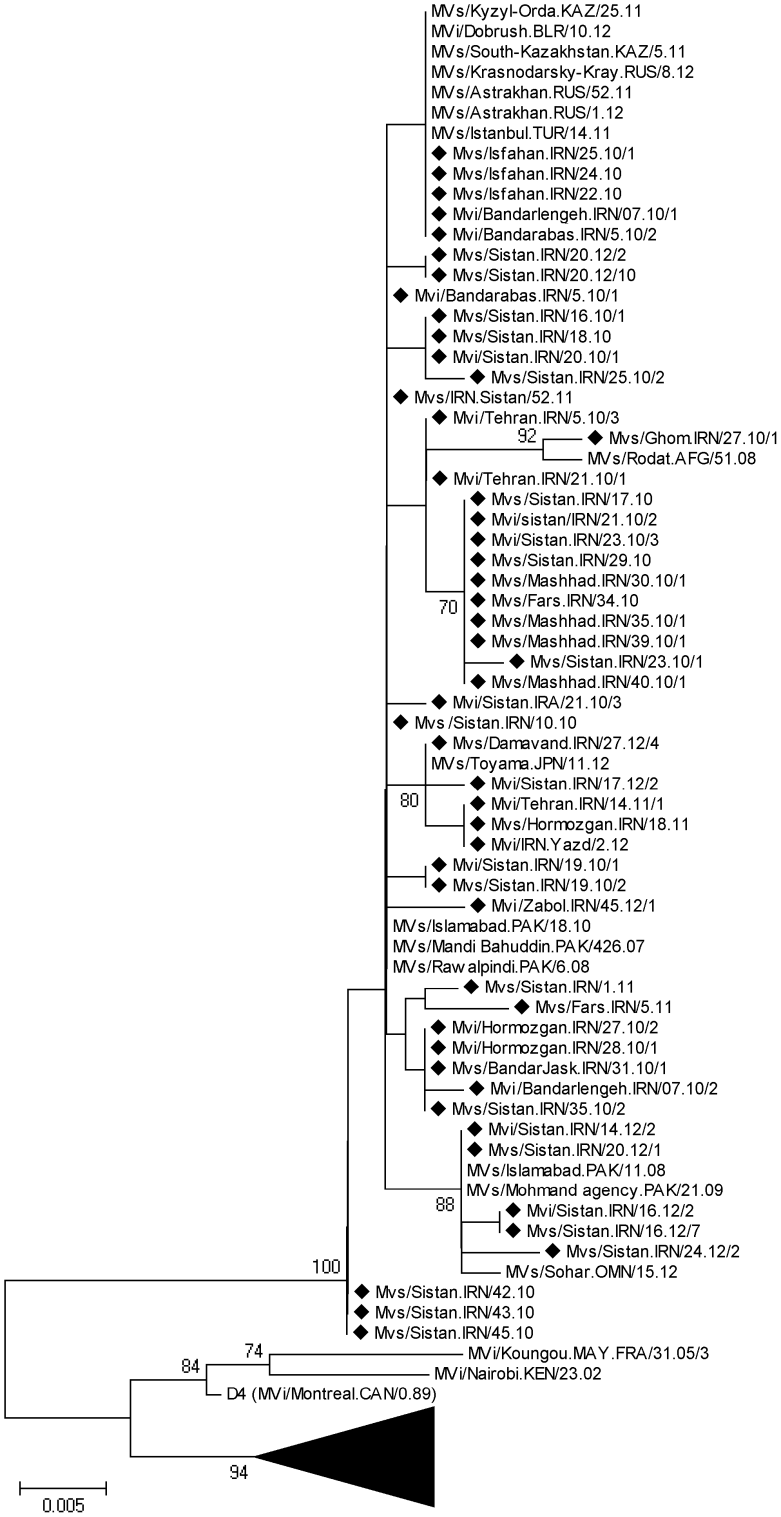
Phylogenetic comparison of 51 selected Iranian D4 strains (C-terminus of N gene) during 2010–2012 with the D4 reference sequence and other wild-type measles genotype D4 detected in other countries. Bootstrap values (1,000 replicates) >70% are indicated. The Iranian sequences are shown by small dark rhombic. Other wild-type measles genotype D4 detected in other countries that are not closely related to the Iranian D4 strains are compressed and shown by dark triangle. Scale bar indicates base substitutions per site.

**Figure 5:**
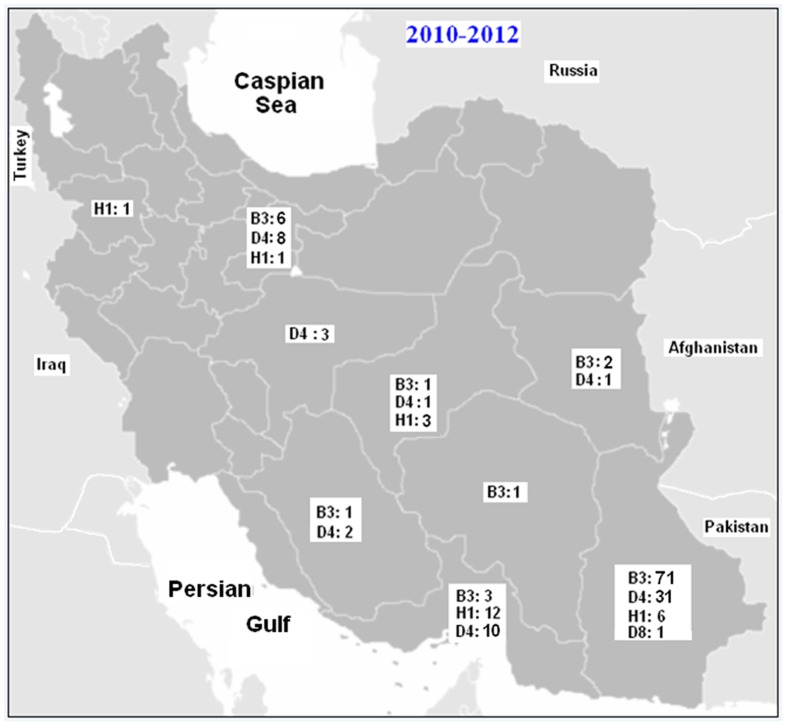
Geographical distribution of measles virus genotypes in Iran (1^st^ May 2010–30^th^ December 2012). Numbers and type of genotypes are shown in boxes.

Sequencing results revealed that 64 (37%) out of 173 samples belonged to genotype D4 (Figure 3). The average sequence identity of 2010–2012 Iranian D4 genotypes was 94.1% and 96.3% with the reference genotypes A and D4, respectively (Edmonston-wt.USA/54 and Montreal.CAN/89). The intra-sequence comparison of these strains ranged from 98.4% to 100% (average 99.4%). One strain (MVs/Ghom.IRN/27.10/1) has 1.3% differences with other strains; the rest had 0.6% differential sequences. These data showed that the source of genotype D4 was not similar in all regions in 2010 because of multiple introductions of different D4 variants (Figure 4). All of the sequences were compared with the sequences previously submitted to GenBank and MeaNS from different parts of the world (Figure 4). The Iranian D4 genotypes are more closely related (99.3–100% identity) to wild-type MVes from Pakistan which were circulating in 2007 (MVs/MandiBahuddin.PAK/426.07). Two previous D4 strain in 2008 (MVs/Khorasan.Iran/20.08 and MVi/Mazandaran.Iran/10.08) in Iran had also 100% identity with 2010 detected strains in Iran (MVi/Bandarabas.IRN/5.10/1, MVs/Sistan.IRA/10.10) and Pakistan (MVs/MandiBahuddin.PAK/426.07). The results documented the continued circulation of genotypes D4 viruses throughout the south, east and center of Iran (Figure 4, 5). The MVi/Bandarabas.IRN/5.10/2 strain also has 100% identity to measles isolates reported from Turkey (MVs/Istanbul.TUR/14.11), Russia (MVs/Astrakhan.RUS/52.11, MVs/Krasnodarsky-Kray.RUS/8.12), Kazakhstan (MVs/South-Kazakhstan.KAZ/5.11) and Belarus (MVi/Dobrush.BLR/10.12) during 2011–2012. It raises this probability that the source of all were the same strain probably from Iran or Pakistan (Figure 4). The MVs/Ghom.IRN/27.10/1 strain has more than 99.5% identity with wild-type MVes from Afghanistan which were circulating in 2008 (MVs/Rodat.AFG/861.08). Also demographic information showed that two Iranian measles cases were originally Pakistanian (MVi/Bandarlengeh.IRN/07.10/1, MVi/Bandarlengeh.IRN/07.10/2) and three were Afghanistanian (MVs/Tehran.IRN/14.10, MVs/Sistan.IRN/42.10, MVs/Sistan.IRN/43.10) who were traveled to our country during 2010 (Table S1). BLAST analysis revealed that the MVs/IRN.Sistan/52.11 strain was 100% identical to the MVi/Bandarabas.IRN/5.10/1 strain, circulating during 2010. Our data confirmed that susceptible persons were still present in 2011 (Figure 4, 5). The MVi/Sistan.IRN/14.12/2 and MVs/Sistan.IRN/20.12/1 are 100% identical to wild-type MVes from Pakistan (MVs/Islamabad.PAK/11.08) (Figure 4, 5).

### Phylogenetic Analysis of MV D8 Genotype

1 out of 173 samples belonged to genotype D8 (Figure 3). The average sequence identity of Iranian genotype D8 strain (Mvs/Sistan.IRN/23.10/2) to the reference genotypes A and D8 (Edmonston-wt.USA/54, Manchester.UNK/30.94) was 94.4% and 97.7%, respectively. BLAST analysis of partial sequences of the N gene confirmed that it was 99.7% identical to the Indian isolates, circulating during 2010 (MVs/PUNE.IND/16.10/1). The patients infected with MV D8 had no history of traveling abroad and their transmission pathway could not be traced (Figure 3).

### Phylogenetic Analysis of MV B3 Genotypes

**Figure 6:**
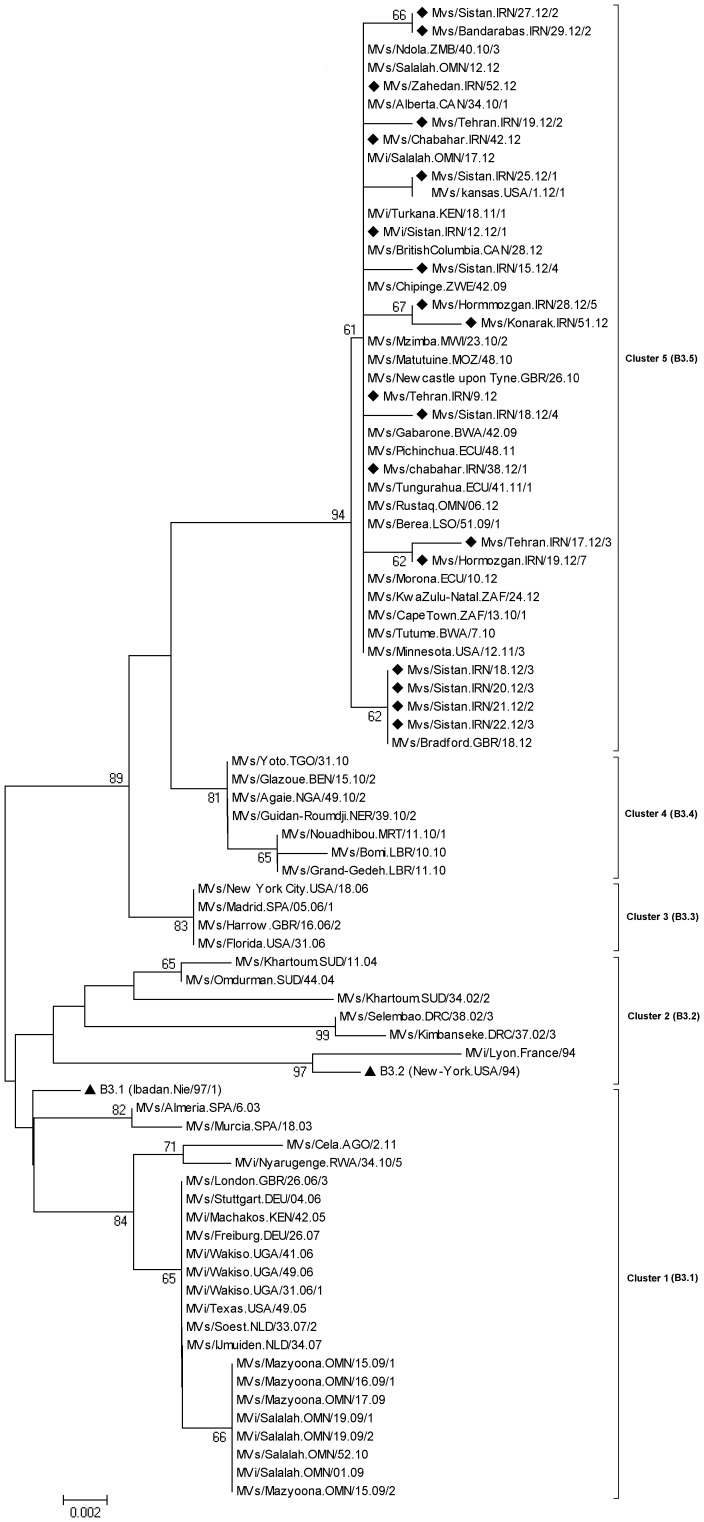
Phylogenetic analysis of 19 selected Iranian B3 strains (C-terminus of N gene) during 2010–2012 with the B3 reference sequence and other wild-type measles genotype B3 detected in other countries. Bootstrap values (1,000 replicates) >70% are indicated. Iranian strains are indicated by dark rhombic. Reference strains are shown by dark triangles. Scale bar indicates base substitutions per site.

Unfortunately the measles outbreaks intensified and continued to occur in 2012 in Iran. During the period from 1^st^ January through 30^th^ December 2012, 85 (49.13%) samples were belonged to genotype B3. 89.4% of these cases were belong to the south, east and south east of Iran where neighbor to the countries that have high measles prevalence (e.g. Pakistan and Afghanistan) (Figure 5). This is the first detection of genotype B3 viruses in Iran. The average sequence identity of Iranian genotypes B3 were 92.2%, 98% and 96.8% with the reference genotypes A, B3.1 and B3.2, respectively (Edmonston-wt.USA/54, Ibadan.Nie/97/1 and New-York.USA/94). Although these strains belonged to the genotype B3, phylogenetic analysis showed that they were quite divergent and located in separate clusters. As demonstrated in Figure 6 within B3 sequences at least five clusters could be distinguished. Iranian B3 prototypes and some other countries sequences were located in the cluster five (Figure 6). BLAST and phylogenetic analysis of partial sequences of the N gene confirmed that most of Iranian B3 genotype (61 strains) were 100% identical to the South African B3 (Cape Town) isolates, circulated during 2010 (e.g. MVs/Cape Town.ZAF/13.10.1) (Figure 6). According to our result, B3 strain (MVi/Sistan.IRN/12.12/2) was isolated form a Pakistani who had traveled to Iran. Some of Iranian B3 genotypes (9 strains) were 100% identical to the UK isolate that was reported at the same week in 2012 (MVs/Bradford.GBR/18.12). Also both are closely related (99.7% identity) to South African B3 (Cape Town) isolate, which circulated during 2010 (e.g. MVs/Cape Town.ZAF/13.10.1) (Figure 6). BLAST and phylogenetic analysis of the rest Iranian B3 genotype (11 strains) revealed that they were also closely related (99.7% identity) to South African B3 genotype (Cape Town) (Figure 6). In addition, the Iranian B3 strain i.e. MVs/Sistan.IRN/25.12/1 was 100% identical to the USA isolate that was reported at the first week in 2012 (MVs/Kansas.USA/1.12/1). Both isolates are closely related (99.7% identity) to South African B3 (Cape Town) isolate (e.g. MVs/Cape Town.ZAF/13.10.1) (Figure 6). The Intra-sequence comparison of Iranian B3 strains ranged from 99.5% to 100% (The average was 99.85%). It shows that the source of many of them might be a single chain of transmission.

### Phylogenetic Analysis of MV H1 Genotypes

**Figure 7:**
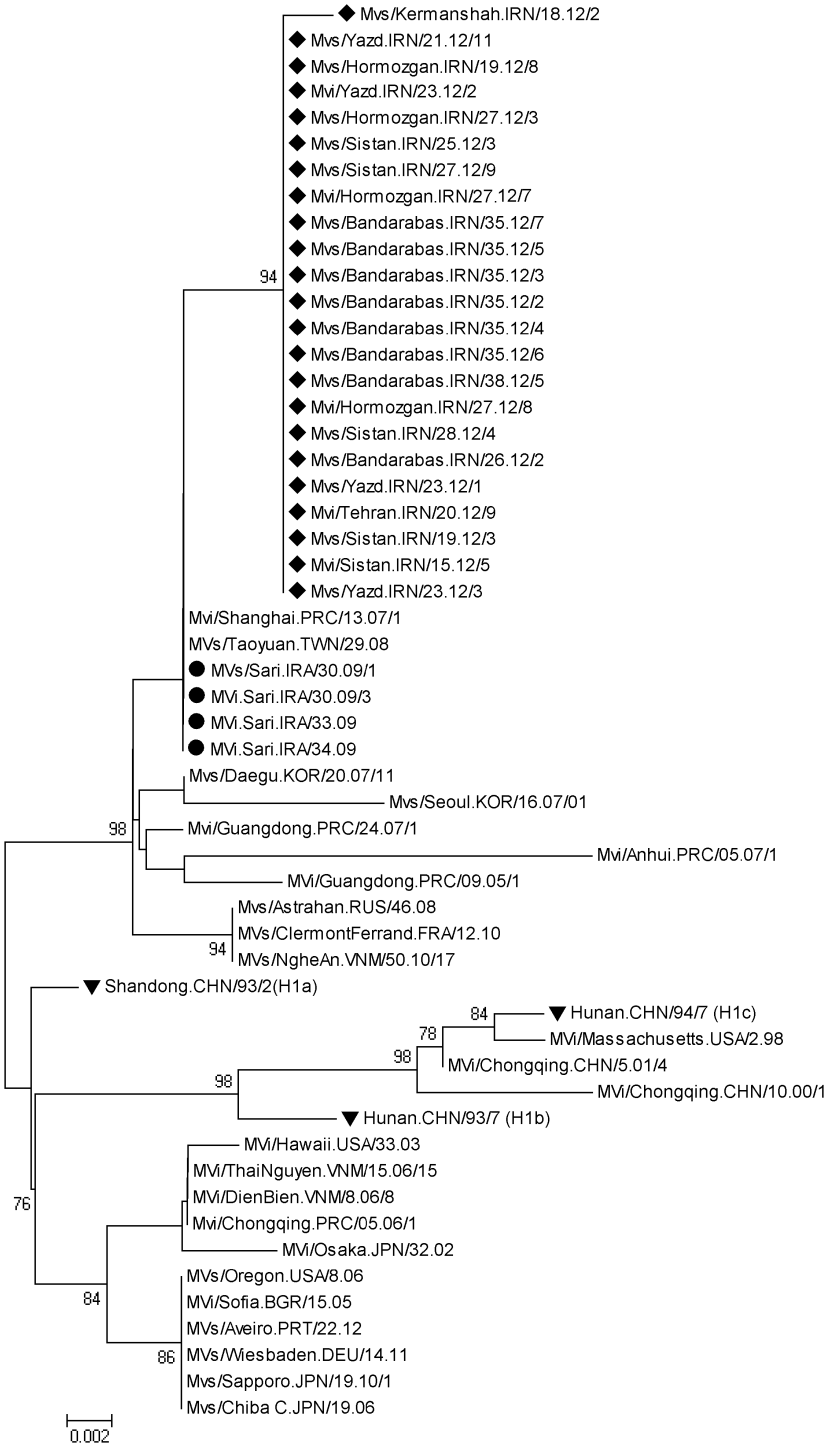
Phylogenetic analysis of all Iranian H1 strains (C-terminus of N gene) during 2010–2012 with the H1 reference sequence and other wild-type measles genotype H1 detected in other countries. Bootstrap values (1,000 replicates) >70% are indicated. 2012- Iranian H1 strains are indicated by dark rhombic. 2009- Iranian H1 strains are indicated by dark circle. Reference strains are shown by dark triangles. Scale bar indicates base substitutions per site.

According to the previous study, the genotype H1 was probably linked to the MV importation from distant parts of Asia in 2009 [7]. 23 (13.3%) out of 173 samples belonged to genotype H1 (Figure 7). Our data showed that the average sequence identity of recent Iranian H1 genotypes were 91.5% and 97.1% with the reference genotypes A and H1, respectively (Edmonston-wt.USA/54 and Hunan.CHN/93/7). BLAST analysis indicated that recent Iranian H1 genotypes were closely related to the Taiwan and China and isolates (MVs/Taoyuan.TWN/29.08 and Mvi/Shanghai.PRC/13.07/1) (Figure 7). The Intra-sequence comparison of recent Iranian H1 strains ranged from 99.7% to 100% (The average was 99.97%). It should be noted that although both genotypes H1 detected in 2009 and 2012 were closely related (99.5% identity) to each other but there is no strong data to confirm that it was circulated from 2009 in Iran (Figure 7).

## Discussion

According to the appropriate sample collection of measles suspected cases as well as serological and molecular assays, genotype information is always available in Iran [5], [7], [8]. After mass vaccination campaign in 2003, there were limited numbers of measles outbreaks in Iran from 2004 to 2008 [5], [6]. However, measles continued to be circulated in Iran and there were several small and large outbreaks during 2009–2012 and most of them occurred in east, south and south east, specially in rural and hard to reach areas (CDC-Iran data). The largest outbreaks in Iran occurred in 2012. Genotyping showed that the most frequently detected genotype was genotype B3 which appeared to be the major strain in the center, south, east and south-east of Iran (Figure 5). Given the enough of data from previous studies in Iran it is completely obvious that the genotype B3 viruses were introduced by recent importation and were not endemic in Iran. This observation is important because the B3 genotype has never been previously reported in Iran. Detection of the same B3 strains from Iran (MVs/Sistan.IRN/18.12/3 and Mvs/Sistan.IRN/25.12/1), UK (MVs/Bradford.GBR/18.12) and USA (MVs/Kansas.USA/1.12/1) suggested simultaneous importations. Genotype B3 is widely distributed throughout Africa [15]. However; sequence identity alone is not proof for importation from African regions, especially when identical sequences have been detected in different parts of the world.

Previous studies differentiated B3 genotype to 3 different clusters [15]. Based on our sequence analysis, we present 5 clusters to differentiate measles virus within B3 (Figure 6). Our epidemiological data did not identify a link between Iranian cases with African B3 strains, indicating that how quickly MV spreads between countries among susceptible individuals and become widely disseminated in a community with high vaccination coverage [16]. Measles transmission can simply occur anywhere, and Iran is not exception of this scenario. It was previously shown that the genotype B3 viruses that were detected in a large outbreak in Africa in 2005 were soon detected throughout the United States and Europe [17]. There is one known case of importation from Pakistan with B3 genotype in week twelve 2012 at the same time of detecting B3 genotype in Iran, rise the probability of circulation of B3 in neighboring countries. Because of proper molecular surveillance in Iran, viruses that were indistinguishable from the neighbor countries were isolated during the outbreak of measles in Iran during 2012.

Genotype H1 was detected in 2009 [7] but there had been no reported cases of measles H1 until 2012. Our phylogenetic analysis has shown that the genotype H1 detected in 2012 was different from those detected in 2009 with 0.5% difference. It seems that genetic heterogeneity of the Iranian H1 strains is not a result of increased mutation rates but is due to re-importation from South-East Asian countries (Figure 5,7).

Although circulation of D4 strains have been documented before 2003 [8], [9] and continued in the study period, the D4 genotype was found circulated only in 7 provinces during 2010–2011 and 4 provinces in 2012, which indicates the limited circulation of D4 in the country (Figure 5). However, it cannot be concluded whether identical or very similar D4 viruses were frequently re-introduced or whether they were continuously circulating and cases were missed. Genetic heterogeneity of D4 genotype in Iran might be due to multiple chains of transmission of this virus that is similar to circulation of the dominant D8 genotype in India [18]. Neighboring countries Pakistan, Afghanistan and Iraq have more severe problems with measles and are therefore likely sources of importations of D4 viruses. Sequence information is not regularly available in Afghanistan and Pakistan. Therefore the sources of genotypes are not apparent in these countries. The borders between Iran, Afghanistan and Pakistan are long and porous. Every day, some groups of people cross over these borders which may have brought different measles genotypes along with them into Iran. On the other hand, if the fraction of susceptible population gradually expands then the probability of a large measles outbreak will increase. The figures 1,2 and 7 shows that although vaccination program is currently good enough to prevent nationwide epidemics and successfully decreased measles incidence in Iran, the fraction of protected individuals in the population was not high enough to prevent continuous introduction of cases from abroad. This problem was also reported from Central African Republic and Taiwan [19], [20]. In the case of independent sources, our molecular characterizations alone were not able to differentiate between continuous circulation of indigenous virus and multiple introductions from the same source.

Previously in Iran only the genotype D4 has been reported. However a rapid change in pattern of MV strains is observed when the genotypes B3 (70% of the 106 genotyped cases) and H1 were predominant in 2012. The replacement of one prevalent genotype by another is a sign that vaccination coverage was high enough to cut off transmission of the first genotype but not high enough to prevent importation/establishment of another genotype. This phenomenon has also been observed in other countries such as Ireland [21].

Our results have shown that 25.4% of samples were isolated from cell culture which is useful for a more extensive characterization of the strains involved in the outbreaks. 83.2% of clinical samples for MV isolation were collected within less than 7 days after rash onset. Inability to grow virus from cell culture might be due to inappropriate transportation of the samples to the laboratory. Similar success rates were also observed in several studies, which were 12% [22] and 15% [23] yields. The largest proportion of cases occurred in toddlers (≤1 year) and young children (5–14 years). Many of the affected toddlers were younger than the age of the first vaccination (12 month). These observations highlight the importance of achieving high vaccination coverage in both groups (Figure 1). This also raised the question whether the first dose of measles vaccine should be brought down to 9 months again. In addition, gradually elevation of measles infection in vaccinated children confirms vaccination failure in 2–4 and 5–14 age groups in Iran (Figure 1). We believe that vaccination of young school aged children and toddlers can be necessary to promptly control outbreaks in south and south east of Iran and prevent a large reservoir of susceptible persons to avoid new outbreaks. Age distribution of cases divided for macro-areas shows that the susceptible population is currently expanding in South and South-East of Iran (Figure 2). In fact, a shift in the age distribution of cases in South and South-East of Iran could support our idea that the majority of cases occurred in that region is because of difficulties of those populations in being reached by vaccination as well as vaccination failure. However, in order to inform public health decisions how to prevent new measles outbreaks a computational prediction model should be developed.

In conclusion, the result of our phylogenetic study confirmed that transmission of MV is not interrupted until 2012. Although the genotype D4 was the major measles genotype in Iran, our results show that in 2012 it was not dominant in the recent measles outbreaks. The age range of affected individuals in different provinces highlights the diversity of measles transmission patterns in Iran. The changing of MV genotype pattern in Iran could provide valuable information to facilitate measuring the degree of MV elimination in the surrounding countries such as Afghanistan and Pakistan.

## Supporting Information

Table S1 Characteristics of the 173 positive samples collected from 1^st^ May 2010 to 30^th^ December 2012 in Iran. **†**Month. **‡**Throat swab. **§**Pakistanis who are imported cases. **¶**Afghanis who are imported cases.
(DOCX)
