# Supplementary material for: Implementation of a National Measles Elimination Program in Iran: Phylogenetic Analysis of Measles Virus Strains Isolated during 2010–2012 Outbreaks
Source: PLoS One. 2014 Apr 15;9(4):e94846. doi: 10.1371/journal.pone.0094846 (PMC3988093; doi:10.1371/journal.pone.0094846)
Supplement: Table S1 — Characteristics of the 173 positive samples collected from 1st May 2010 to 30th December 2012 in Iran. †Month. ‡Throat swab. §Pakistanis who are imported cases. ¶Afghanis who are imported cases. (DOCX) [file pone.0094846.s001.docx]

**Table S1.** Characteristics of the 173 positive samples collected from 1^st^ May 2010 to 30^th^ December 2012 in Iran

| **NO** | **Strain Designation** | **Age**  **(year)** | **Gender** | **Days after rash onset** | **Source of**  **specimen** | **Genotype** | **Region of origin** | **Vaccine**  **History** | **Accession number** |
| --- | --- | --- | --- | --- | --- | --- | --- | --- | --- |
| 1 | MVi/Bandarabas.IRN/5.10/1 | 9M **†** | F | 6 | Urine | D4 | South | None | HM440226 |
| 2 | MVi/Bandarabas.IRN/5.10/2 | 4M | M | 4 | Urine | D4 | South | None | HM440227 |
| 3 | MVi/Tehran.IRN/5.10/3 | 12 | M | 3 | Urine | D4 | Center | Yes | HM998696 |
| 4 | MVi/Bandarlengeh.IRN/07.10/1 | 15 | F | 1 | Urine | D4 | South **^§^** | Unknown | HM440228 |
| 5 | MVi/Bandarlengeh.IRN/07.10/2 | 27 | M | 3 | Urine | D4 | South **^§^** | Unknown | HM440229 |
| 6 | MVs/Sistan.IRN/10.10 | 4M | F | 5 | TS **^‡^** | D4 | South East | None | HM998697 |
| 7 | MVs/Tehran.IRN/14.10 | 20 | M | 5 | TS | D4 | Center **^¶^** | None | HM998698 |
| 8 | MVs/Sistan.IRN/16.10/1 | 2 | F | 7 | TS | D4 | South East | Yes | HM998699 |
| 9 | MVi/Sistan.IRN/16.10/2 | 2 | M | 6 | TS | D4 | South East | None | HM998700 |
| 10 | MVs/Sistan.IRN/17.10 | 6M | M | 4 | TS | D4 | South East | None | HM998701 |
| 11 | MVs/Sistan.IRN/18.10 | 4 | M | 4 | TS | D4 | South East | None | HM998702 |
| 12 | MVi/Sistan.IRN/19.10/1 | 4 | M | 10 | TS | D4 | South East | Yes | HM998703 |
| 13 | MVs/Sistan.IRN/19.10/2 | 7M | M | 11 | TS | D4 | South East | Yes | HM998704 |
| 14 | MVi/Sistan.IRN/20.10/1 | 2 | M | 8 | TS | D4 | South East | Yes | HM998705 |
| 15 | MVs/Sistan.IRN/20.10/2 | 4 | F | 1 | TS | D4 | South East | Yes | HM998706 |
| 16 | MVi/Tehran.IRN/21.10/1 | 23 | M | 3 | Urine | D4 | Center | None | HM998707 |
| 17 | MVi/Sistan.IRN/21.10/2 | 11 | M | 3 | TS | D4 | South East | Unknown | HM998708 |
| 18 | MVi/Sistan.IRN/21.10/3 | 2 | F | 5 | TS | D4 | South East | None | HM998709 |
| 19 | MVs/Isfahan.IRN/22.10 | 5 | M | 2 | TS | D4 | Center | Yes | HQ395674 |
| 20 | MVs/Sistan.IRN/23.10/1 | 1 | F | 3 | TS | D4 | South East | None | HQ395675 |
| 21 | MVs/Sistan.IRN/23.10/2 | 2 | F | 5 | TS | D8 | South East | None | HQ395676 |
| 22 | MVi/Sistan.IRN/23.10/3 | 5 | M | 4 | TS | D4 | South East | None | HQ596516 |
| 23 | MVs/Isfahan.IRN/24.10 | 3 | M | 3 | TS | D4 | Center | Yes | HQ395677 |
| 24 | MVs/Isfahan.IRN/25.10/1 | 8M | M | 5 | TS | D4 | Center | None | HQ395678 |
| 25 | MVs/Sistan.IRN/25.10/2 | 2 | F | 9 | TS | D4 | South East | Yes | HQ395679 |
| 26 | MVs/Ghom.IRN/27.10/1 | 16 | M | 3 | TS | D4 | Center | Unknown | HQ395680 |
| 27 | MVi/Hormozgan.IRN/27.10/2 | 9 | F | 7 | TS | D4 | South | Yes | HQ596505 |
| 28 | MVi/Hormozgan.IRN/28.10/1 | 31 | F | 6 | Urine | D4 | South | Unknown | HQ596506 |
| 29 | MVi/Hormozgan.IRN/28.10/2 | 3 | M | 6 | Urine | D4 | South | Yes | HQ596507 |
| 30 | MVs/Sistan.IRN/29.10 | 11M | M | 4 | TS | D4 | South East | None | HQ395681 |
| 31 | MVs/Mashhad.IRN/30.10/1 | 8M | F | 2 | TS | D4 | North East | None | HQ395682 |
| 32 | MVi/Mashhad.IRN/30.10/2 | 3 | F | 3 | Urine | D4 | North East | Unknown | HQ395683 |
| 33 | MVs/Mashhad.IRN/30.10/3 | 34 | F | 3 | TS | D4 | North East | Unknown | HQ596508 |
| 34 | MVs/Mashhad.IRN/30.10/4 | 1 | F | 5 | TS | D4 | North East | None | HQ596509 |
| 35 | MVs/BandarJask.IRN/31.10/1 | 6 | F | 3 | TS | D4 | South | Yes | HQ395684 |
| 36 | MVs/BandarJask.IRN/31.10/2 | 6 | F | 3 | TS | D4 | South | Yes | HQ395685 |
| 37 | MVs/Fars.IRN/34.10 | 11M | F | 3 | TS | D4 | South West | Unknown | HQ596510 |
| 38 | MVs/Mashhad.IRN/35.10/1 | 8 | F | 1 | Urine | D4 | North East | Yes | HQ596511 |
| 39 | MVs/Sistan.IRN/35.10/2 | 11M | F | 1 | TS | D4 | South East | Yes | HQ596512 |
| 40 | MVs/Sistan.IRN/35.10/3 | 13 | F | 8 | Urine | D4 | South East | Unknown | HQ596513 |
| 41 | MVs/Mashhad.IRN/39.10/1 | 8M | M | 4 | TS | D4 | North East | Unknown | HQ596514 |
| 42 | MVs/Mashhad.IRN/39.10/2 | 8M | M | 4 | TS | D4 | North East | Unknown | HQ596515 |
| 43 | MVs/Mashhad.IRN/40.10/1 | 10M | M | 3 | TS | D4 | North East | Unknown | HQ668019 |
| 44 | MVs/Sistan.IRN/40.10/2 | 2 | F | 7 | TS | D4 | South East | Yes | HQ668020 |
| 45 | MVs/Sistan.IRN/42.10 | 1M | M | 9 | TS | D4 | South East **^¶^** | Unknown | HQ668021 |
| 46 | MVs/Sistan.IRN/43.10 | 2 | M | 8 | TS | D4 | South East **^¶^** | Unknown | HQ668022 |
| 47 | MVs/Sistan.IRN/45.10 | 2 | M | 4 | TS | D4 | South East | Unknown | HQ711619 |
| 48 | MVs/Sistan.IRN/1.11 | 7 | F | 6 | TS | D4 | South East | Unknown | JF327854 |
| 49 | MVs/Fars.IRN/5.11 | 7 | F | 2 | TS | D4 | South West | None | JF716447 |
| 50 | MVi/Tehran.IRN/14.11/1 | 21 | M | 4 | Urine | D4 | Center | Unknown | JN048663 |
| 51 | MVs/Tehran.IRN/14.11/2 | 21 | M | 5 | Urine | D4 | Center | Unknown | JN048664 |
| 52 | MVs/Hormozgan.IRN/18.11 | 1 | F | 1 | Urine | D4 | South | None | JN048665 |
| 53 | MVs/Sistan.IRN/52.11 | 7 | F | 2 | Urine | D4 | South East | Unknown | JQ687376 |
| 54 | MVi/Yazd.IRN/2.12 | 23 | M | 5 | Urine | D4 | Center | Unknown | JQ687377 |
| 55 | MVs/Tehran.IRN/9.12 | 28 | M | 8 | TS | B3 | Center | Unknown | JX183266 |
| 56 | MVi/Sistan.IRN/12.12/1 | 5 | M | 1 | TS | B3 | South East | Unknown | JX051516 |
| 57 | MVi/Sistan.IRN/12.12/2 | 2 | F | 1 | TS | B3 | South East **^§^** | Unknown | JX051517 |
| 58 | MVs/Sistan.IRN/14.12/1 | 17 | F | 8 | TS | B3 | South East | None | JX183259 |
| 59 | MVi/Sistan.IRN/14.12/2 | 6M | M | 9 | Urine | D4 | South East | None | JX183260 |
| 60 | MVi/Sistan.IRN/15.12/1 | 11 | M | 2 | Urine | B3 | South East | None | JX183261 |
| 61 | MVi/Sistan.IRN/15.12/2 | 1 | M | 1 | Urine | B3 | South East | Unknown | JX183262 |
| 62 | MVi/Sistan.IRN/15.12/3 | 1 | M | 5 | TS | B3 | South East | None | JX183263 |
| 63 | MVs/Sistan.IRN/15.12/4 | 11M | M | 8 | TS | B3 | South East | None | JX183264 |
| 64 | MVi/Sistan.IRN/15.12/5 | 24 | M | 3 | TS | H1 | South East | Unknown | JX183265 |
| 65 | MVi/Sistan.IRN/16.12/1 | 5 | M | 1 | TS | B3 | South East | Yes | JX183267 |
| 66 | MVi/Sistan.IRN/16.12/2 | 28 | M | 1 | TS | D4 | South East | None | JX183268 |
| 67 | MVi/Sistan.IRN/16.12/3 | 3 | F | 2 | TS | B3 | South East | None | JX183269 |
| 68 | MVs/Sistan.IRN/16.12/4 | 1 | F | 9 | TS | B3 | South East | None | JX183270 |
| 69 | MVs/Sistan.IRN/16.12/5 | 1 | M | 11 | TS | B3 | South East | Yes | JX183271 |
| 70 | MVs/Sistan.IRN/16.12/6 | 1 | M | 8 | TS | B3 | South East | None | JX183272 |
| 71 | MVs/Sistan.IRN/16.12/7 | 2 | M | 1 | TS | D4 | South East | Unknown | JX183273 |
| 72 | MVi/Sistan.IRN/17.12/1 | 11M | M | 2 | TS | B3 | South East | None | JX183274 |
| 73 | MVi/Sistan.IRN/17.12/2 | 1 | M | 5 | TS | D4 | South East | None | JX183275 |
| 74 | MVs/Tehran.IRN/17.12/3 | 1.6 | F | 5 | Urine | B3 | Center | None | JX219969 |
| 75 | MVs/Tehran.IRN/18.12/1 | 3 | M | 7 | Urine | B3 | Center | None | JX183276 |
| 76 | MVs/Kermanshah.IRN/18.12/2 | 14 | M | 4 | TS | H1 | West | Yes | JX183277 |
| 77 | MVs/Sistan.IRN/18.12/3 | 6 | F | 6 | TS | B3 | South East | Yes | JX219957 |
| 78 | MVs/Sistan.IRN/18.12/4 | 1 | M | 7 | Urine | B3 | South East | None | JX219958 |
| 79 | MVs/Sistan.IRN/18.12/5 | 1 | F | 2 | TS | B3 | South East | None | JX219959 |
| 80 | MVi/Sistan.IRN/18.12/6 | 6M | F | 1 | Urine | B3 | South East | None | JX219960 |
| 81 | MVi/Sistan.IRN/19.12/1 | 10 | M | 4 | Urine | B3 | South East | Unknown | JX219961 |
| 82 | MVs/Tehran.IRN/19.12/2 | 20 | M | 3 | TS | B3 | Center | Unknown | JX219962 |
| 83 | MVs/Sistan.IRN/19.12/3 | 8M | F | 3 | TS | H1 | South East | None | JX219963 |
| 84 | MVs/Sistan.IRN/19.12/4 | 8 | M | 2 | TS | B3 | South East | Unknown | JX219964 |
| 85 | MVi/Sistan.IRN/19.12/5 | 7 | F | 6 | TS | B3 | South East | None | JX219965 |
| 86 | MVs/Sistan.IRN/19.12/6 | 9 | M | 9 | TS | B3 | South East | None | JX219966 |
| 87 | MVs/Hormozgan.IRN/19.12/7 | 4 | M | 3 | TS | B3 | South | Unknown | JX219967 |
| 88 | MVs/Hormozgan.IRN/19.12/8 | 1 | F | 4 | TS | H1 | South | None | JX418023 |
| 89 | MVs/Sistan.IRN/20.12/1 | 5 | M | 1 | TS | D4 | South East | None | JX219968 |
| 90 | MVs/Sistan.IRN/20.12/2 | 12 | F | 4 | TS | D4 | South East | Unknown | JX266440 |
| 91 | MVs/Sistan.IRN/20.12/3 | 14 | M | 3 | TS | B3 | South East | Unknown | JX266441 |
| 92 | MVs/Sistan.IRN/20.12/4 | 5M | M | 4 | TS | B3 | South East | None | JX266442 |
| 93 | MVs/Sistan.IRN/20.12/5 | 1 | F | 3 | TS | B3 | South East | Unknown | JX266443 |
| 94 | MVs/Sistan.IRN/20.12/6 | 10M | F | 4 | TS | B3 | South East | None | JX266444 |
| 95 | MVs/Sistan.IRN/20.12/7 | 6M | F | 2 | TS | B3 | South East | None | JX266445 |
| 96 | MVs/Sistan.IRN/20.12/8 | 1 | M | 1 | TS | B3 | South East | None | JX418024 |
| 97 | MVi/Tehran.IRN/20.12/9 | 24 | M | 1 | Urine | H1 | Center | Unknown | JX266446 |
| 98 | MVs/Sistan.IRN/20.12/10 | 6 | M | 3 | TS | D4 | South East | Unknown | JX418025 |
| 99 | MVs/Sistan.IRN/20.12/11 | 7M | F | 5 | TS | B3 | South East | None | JX418026 |
| 100 | MVs/Tehran.IRN/21.12/1 | 33 | F | 3 | TS | B3 | Center | Unknown | JX418027 |
| 101 | MVs/Sistan.IRN/21.12/2 | 10M | F | 5 | TS | B3 | South East | None | JX418028 |
| 102 | MVs/Sistan.IRN/21.12/3 | 7 | M | 2 | TS | B3 | South East | Unknown | JX418029 |
| 103 | MVs/Sistan.IRN/21.12/4 | 1 | F | 4 | TS | B3 | South East | Unknown | JX418030 |
| 104 | MVs/Sistan.IRN/21.12/5 | 11M | F | 6 | Urine | B3 | South East | Unknown | JX418031 |
| 105 | MVs/Sistan.IRN/21.12/6 | 2 | M | 6 | TS | B3 | South East | None | JX434636 |
| 106 | MVs/Sistan.IRN/21.12/7 | 1 | M | 7 | TS | B3 | South East | None | JX486007 |
| 107 | MVs/Sistan.IRN/21.12/8 | 23 | M | 15 | Urine | B3 | South East | Unknown | JX486008 |
| 108 | MVs/Fars.IRN/21.12/9 | 1 | M | 6 | TS | B3 | South West | None | JX631242 |
| 109 | MVs/Sistan.IRN/21.12/10 | 2 | M | 5 | TS | B3 | Center | Unknown | JX857301 |
| 110 | MVs/Yazd.IRN/21.12/11 | 18 | M | 6 | TS | H1 | South East | Unknown | JX631243 |
| 111 | MVi/Sistan.IRN/22.12/1 | 11M | M | 4 | TS | B3 | South East | None | JX486009 |
| 112 | MVs/Sistan.IRN/22.12/2 | 3 | F | 11 | TS | B3 | South East | Unknown | JX486010 |
| 113 | MVs/Sistan.IRN/22.12/3 | 3M | M | 5 | TS | B3 | South East | None | JX631244 |
| 114 | MVs/Sistan.IRN/22.12/4 | 7M | M | 15 | TS | B3 | South East | None | JX631245 |
| 115 | MVs/Yazd.IRN/23.12/1 | 22 | M | 10 | TS | H1 | Center | Unknown | JX418032 |
| 116 | MVi/Yazd.IRN/23.12/2 | 12 | M | 3 | Urine | H1 | Center | Unknown | JX486006 |
| 117 | MVs/Yazd.IRN/23.12/3 | 9 | M | 3 | Urine | H1 | Center | None | JX486011 |
| 118 | MVs/Sistan.IRN/23.12/4 | 9M | M | 2 | TS | B3 | South East | Unknown | JX486012 |
| 119 | MVs/Ghom.IRN/24.12/1 | 22 | M | 4 | Urine | B3 | Center | Unknown | JX486013 |
| 120 | MVs/Sistan.IRN/24.12/2 | 11M | F | 5 | TS | D4 | South East | Unknown | JX486014 |
| 121 | MVs/Sistan.IRN/24.12/3 | 10M | F | 5 | TS | B3 | South East | None | JX631246 |
| 122 | MVs/Sistan.IRN/25.12/1 | 14 | M | 9 | Urine | B3 | South East | Yes | JX486015 |
| 123 | MVi/Sistan.IRN/25.12/2 | 6M | F | 4 | TS | B3 | South East | None | JX631247 |
| 124 | MVs/Sistan.IRN/25.12/3 | 4 | M | 6 | Urine | H1 | South East | Yes | JX631248 |
| 125 | MVs/Sistan.IRN/26.12/1 | 10M | M | 4 | TS | B3 | South East | None | JX631249 |
| 126 | MVs/Bandarabas.IRN/26.12/2 | 1 | M | 1 | TS | H1 | South | None | JX631250 |
| 127 | MVs/Sistan.IRN/27.12/1 | 3 | M | 10 | TS | B3 | South East | None | JX631251 |
| 128 | MVs/Sistan.IRN/27.12/2 | 6M | M | 7 | TS | B3 | South East | None | JX631252 |
| 129 | MVs/Hormozgan.IRN/27.12/3 | 5M | M | 3 | TS | H1 | South | None | JX631253 |
| 130 | MVs/Damavand.IRN/27.12/4 | 6 | M | 4 | TS | D4 | North | Unknown | JX631254 |
| 131 | MVs/Sistan.IRN/27.12/5 | 5 | M | 5 | TS | B3 | South East | Unknown | JX631255 |
| 132 | MVs/Damavand.IRN/27.12/6 | 4 | F | 5 | TS | D4 | North | Unknown | JX857302 |
| 133 | MVi/Hormozgan.IRN/27.12/7 | 40 | F | 2 | Urine | H1 | South | Unknown | JX857303 |
| 134 | MVi/Hormozgan.IRN/27.12/8 | 60 | F | 5 | Urine | H1 | South | Unknown | JX857304 |
| 135 | MVs/Sistan.IRN/27.12/9 | 10M | M | 10 | TS | H1 | South East | None | JX857305 |
| 136 | MVs/Sistan.IRN/27.12/10 | 6 | M | 6 | TS | B3 | South East | None | JX857306 |
| 137 | MVs/Sistan.IRN/28.12/1 | 6M | F | 4 | TS | B3 | South East | None | JX857307 |
| 138 | MVs/Sistan.IRN/28.12/2 | 4 | M | 4 | TS | B3 | South East | Unknown | JX857308 |
| 139 | MVs/Sistan.IRN/28.12/3 | 8 | F | 6 | TS | B3 | South East | Unknown | JX857309 |
| 140 | MVs/Sistan.IRN/28.12/4 | 1 | M | 7 | TS | H1 | South East | None | JX857310 |
| 141 | MVs/Hormmozgan.IRN/28.12/5 | 6 | M | 8 | TS | B3 | South | Yes | JX857311 |
| 142 | MVs/Kerman.IRN/29.12/1 | 10 | M | 2 | TS | B3 | Center | Yes | JX857312 |
| 143 | MVs/Bandarabas.IRN/29.12/2 | 31 | M | 1 | TS | B3 | South | None | JX857313 |
| 144 | MVi/Birjand.IRN/29.12/3 | 6M | F | 4 | TS | B3 | East | None | KC139050 |
| 145 | MVi/Birjand.IRN/29.12/4 | 10M | F | 4 | TS | B3 | East | None | KC139051 |
| 146 | MVs/Sistan.IRN/30.12/1 | 2 | M | 7 | TS | B3 | South East | None | JX857314 |
| 147 | MVs/Sistan.IRN/30.12/2 | 10M | M | 5 | TS | B3 | South East | Yes | JX857315 |
| 148 | MVs/Konarak.IRN/35.12/1 | 6M | M | 10 | Urine | B3 | South East | None | KC139052 |
| 149 | MVs/Bandarabas.IRN/35.12/2 | 10 | M | 1 | TS | H1 | South | Yes | KC139053 |
| 150 | MVs/Bandarabas.IRN/35.12/3 | 7 | F | 2 | Urine | H1 | South | Yes | KC139054 |
| 151 | MVs/Bandarabas.IRN/35.12/4 | 11 | M | 2 | TS | H1 | South | Yes | KC139055 |
| 152 | MVs/Bandarabas.IRN/35.12/5 | 9 | M | 2 | Urine | H1 | South | Yes | KC139056 |
| 153 | MVs/Bandarabas.IRN/35.12/6 | 1 | M | 3 | TS | H1 | South | None | KC139057 |
| 154 | MVs/Bandarabas.IRN/35.12/7 | 7 | M | 2 | TS | H1 | South | Yes | KC139058 |
| 155 | MVs/Chabahar.IRN/38.12/1 | 2.4 | M | 12 | TS | B3 | South East | Yes | KC139059 |
| 156 | MVs/Chabahar.IRN/38.12/2 | 2.2 | M | 5 | TS | B3 | South East | Unknown | KC139060 |
| 157 | MVs/Chabahar.IRN/38.12/3 | 7M | F | 4 | TS | B3 | South East | None | KC139061 |
| 158 | MVs/Chabahar.IRN/38.12/4 | 1.4 | F | 5 | TS | B3 | South East | None | KC139062 |
| 159 | MVs/Bandarabas.IRN/38.12/5 | 2 | F | 3 | TS | H1 | South | Yes | KC139063 |
| 160 | MVi/Chabahar.IRN/38.12/6 | 7 | M | 7 | Urine | B3 | South East | None | KC879272 |
| 161 | MVi/Chabahar.IRN/38.12/7 | 2 | F | 6 | Urine | B3 | South East | None | KF280869 |
| 162 | MVs/Chabahar.IRN/42.12 | 1.2 | M | 15 | TS | B3 | South East | None | KC879273 |
| 163 | MVs/Zahedan.IRN/45.12/2 | 1.5 | M | 3 | TS | B3 | South East | Yes | KF214766 |
| 164 | MVs/Chabahar.IRN/45.12/3 | 7M | M | 4 | TS | B3 | South East | None | KF214767 |
| 165 | MVs/Chabahar.IRN/45.12/4 | 9M | M | 11 | TS | B3 | South East | None | KF214768 |
| 166 | MVs/Chabahar.IRN/45.12/5 | 1.1 | F | 9 | Urine | B3 | South East | None | KF214769 |
| 167 | MVs/Iranshahr.IRN/43.12/1 | 1.2 | F | 5 | TS | B3 | South East | None | KF214765 |
| 168 | MVi/Zabol.IRN/45.12/1 | 7 | M | 3 | Urine | D4 | East | Yes | KC984305 |
| 169 | MVs/Chabahar.IRN/50.12/1 | 10M | M | 12 | TS | B3 | South East | None | KC737545 |
| 170 | MVs/Chabahar.IRN/50.12/2 | 4 | F | 5 | TS | B3 | South East | None | KC737546 |
| 171 | MVs/Chabahar.IRN/50.12/3 | 9M | M | 4 | TS | B3 | South East | None | KC794527 |
| 172 | MVs/Konarak.IRN/51.12 | 9M | M | 3 | TS | B3 | South East | None | KC794528 |
| 173 | MVs/Zahedan.IRN/52.12 | 1M | M | 3 | TS | B3 | South East | None | KC794532 |

**†** Month.

**‡** Throat swab.

**§** Pakistanis who are imported cases.

**¶** Afghanis who are imported cases.
